# Impact of Aligners and Fixed Appliances on Oral Health during Orthodontic Treatment: A Systematic Review and Meta-Analysis

**DOI:** 10.3290/j.ohpd.b2403661

**Published:** 2021-12-08

**Authors:** Elissaios Oikonomou, Petros Foros, Aikaterini Tagkli, Christos Rahiotis, Theodore Eliades, Despina Koletsi

**Affiliations:** a Dentist, School of Dentistry, National and Kapodistrian University of Athens, Athens, Greece. Study concept, design and planning, collected data, wrote the manuscript, read and approved the final manuscript.; b Postgraduate Student, Section of Orthodontics, Department of Dentistry and Oral Health, Aarhus University, Aarhus, Denmark. Study concept, design and planning, collected data, wrote the manuscript, read and approved the final manuscript.; c Associate Professor, Department of Operative Dentistry, School of Dentistry, National and Kapodistrian University of Athens, Athens, Greece. Study concept, design and planning, collected data, wrote the manuscript, read and approved the final manuscript.; d Professor and Director, Clinic of Orthodontics and Pediatric Dentistry, Center of Dental Medicine, University of Zürich, Zürich, Switzerland. Wrote the manuscript, read and approved the final manuscript.; e Senior Teaching and Research Staff, Clinic of Orthodontics and Pediatric Dentistry, Center of Dental Medicine, University of Zürich, Zürich, Switzerland. Study concept, design and planning, collected and analysed data, wrote the manuscript, read and approved the final manuscript, project administration.; * Contributed equally to the study.

**Keywords:** fixed appliances, oral hygiene, orthodontic aligners, systematic review

## Abstract

**Purpose:**

To identify and assess differences in oral hygiene parameters in patients undergoing orthodontic treatment with clear aligners compared to fixed appliances.

**Materials and Methods:**

Published and unpublished literature was searched in seven databases until May 31st 2021. Representative keywords included ‘orthodontic aligner’, ‘fixed appliance’, ‘oral hygiene’, ‘plaque index’, ‘caries’. Study selection, data extraction, risk of bias and certainty of evidence assessment were undertaken independently by three reviewers. Random effects meta-analyses with respective confidence intervals (95% CI) were conducted, where applicable.

**Results:**

A total of 882 unique records were screened, with a final number of 21 articles being eligible for qualitative synthesis, while 4 of those contributed to meta-analyses. Risk of bias was rated within the range of low to high or serious overall, while certainty of evidence was low to very low according to GRADE. For periodontal parameters, adults undergoing aligner orthodontic treatment presented summary plaque scores 0.58 lower than those treated with fixed appliances, within the first 6 to 12 weeks (4 studies: mean difference: -0.58; 95%CI: -0.82, -0.34; p < 0.001; I^2^ squared: 71.3%), while no evidence of difference was recorded for inflammation indices. Microbiologic parameters such as presence of *S. mutans* and lactobacilli were more pronounced in patients with fixed appliances for the first 3 to 6 months (synthesised data from 2 studies).

**Conclusions:**

In the short-term after initiation of orthodontic treatment, patients treated with aligners and no additional attachments/adjuncts presented potentially higher levels of oral health overall. However, the evidence is supported by low to very low certainty.

**Supplementary Table 1:** Detailed assessment of RoB 2.0 tool.

Domain	Reference	Abbate et al. 2015	Albhaisi et al. 2020	Chibber et al. 2017	Gujar et al. 2019	Levrini et a. 2013	Levrini et al. 2015
1. Randomization process	1.1	Y	Y	Y	NI	Y	Y
	1.2	NI	PY	Y	NI	NI	NI
	1.3	PN	M	N	N	Y	Y
	Assessor’s Judgement	Low	Low	Low	Low	High	High
2. Deviations from intended interventions	2.1	Y	Y	Y	Y	Y	Y
	2.2	Y	Y	Y	Y	Y	Y
	2.3	N	NA	PN	N	PN	NI
	2.4	NA	NA	NA	NA	NA	NA
	2.5	NA	NA	NA	NA	NA	NA
	2.6	PY	PN	PN	Y	PY	PY
	2.7	NA	NA	NA	NA	NA	NA
	Assessor’s Judgement	Low	Some concerns	Low	Low	Low	Some concerns
3. Mising outcome data	3.1	N	Y	N	Y	PY	NI
	3.2	PN	NA	PN	NA	NA	PN
	3.3	PN	NA	PY	NA	NA	NI
	3.4	NA	NA	PN	NA	NA	NI
	Assessor’s judgement	Low	Low	Some concerns	Low	Low	High
4. Measurement of the outcome	4.1	N	N	N	N	N	N
	4.2	PN	N	N	N	PN	PN
	4.3	NI	N	N	N	PN	PN
	4.4	PY	NA	NA	NA	NA	NA
	4.5	N	NA	NA	NA	NA	NA
	Assessor’s Judgement	Some concerns	Low	Low	Low	Low	Low
5. Selection of the reported result	5.1	Y	Y	Y	Y	PY	PY
	5.2	NI	N	N	N	PN	PN
	5.3	PN	N	N	N	PN	PN
	Assessor’s Judgement	Some concerns	Low	Low	Low	Low	Low
Overall	Assessor’s Judgement	Some concerns	Some concerns	Some concerns	Low	High	High
Note		-	-	-	-	-	-

N, no; NA, not applicable; NI, no information; PN, probably no; PY, probably yes; Y, yes

**Supplementary Table 2:** Detailed assessment of included non-randomized studies with the ROBINS-I tool.

Domain	Reference	Azaripour et al. 2015	Buschang et al. 2018	Dallel et al. 2020	Gujar et al. 2020	Han et al. 2015	Karhanetci et al. 2013	Madariaga et al. 2020	Miethke et al. 2005	Miethke et al. 2007	Mulla Issa et al. 2020	Mummolo et al. 2020a	Mummolo et al. 2020b	Sifakakis et al. 2018	Srinath et al. 2016	Wang et al. 2019
1. Confounding	1.1	Y	Y	Y	Y	Y	PY	Y	Y	Y	Y	Y	Y	Y	Y	Y
	1.2	N	N	N	N	N	N	N	N	N	N	N	N	N	PN	N
	1.3	NA	NA	NA	NA	NA	NA	NA	NA	NA	NA	NA	NA	NA	NA	NA
	1.4	PN	PN	Y	Y	PN	Y	PY	PN	PN	PN	PN	PN	Y	NI	Y
	1.5	NA	NA	Y	Y	NA	Y	PY	NA	NA	NA	NA	NA	Y	NA	Y
	1.6	N	NA	N	N	NA	N	N	NA	NA	NA	NA	NA	N	NI	N
	1.7	PN	PN	NA	Y	PN	Y	NI	PN	PN	PN	PN	PN	Y	NI	Y
	1.8	NA	NA	NA	Y	NA	Y	NA	NA	NA	NA	NA	NA	Y	NA	Y
	Judgement	Serious	Serious	Moderate	Low	Serious	Moderate	NI	Serious	Serious	Serious	Serious	Serious	Moderate	NI	Low
2. Selection of participants into the study	2.1	PY	PY	N	N	PY	N	N	NI	NI	PY	N	N	N	NI	N
	2.2	PY	PY	NA	NA	PY	NA	NA	NA	NA	PY	NA	NA	NA	NI	NA
	2.3	PY	PY	NA	NA	PY	NA	NA	NA	NA	PY	NA	NA	NA	NI	NA
	2.4	Y	Y	Y	Y	Y	Y	PN	NI	NI	Y	Y	Y	Y	PY	Y
	2.5	PN	PN	NA	NA	PN	NA	NI	NA	NA	PN	NA	NA	NA	NI	NA
	Judgement	Serious	Serious	Low	Low	Serious	Low	NI	NI	NI	Serious	Low	Low	Low	NI	Low
3. Classification of interventions	3.1	Y	Y	Y	Y	Y	Y	Y	NI	NI	Y	Y	Y	Y	Y	Y
	3.2	Y	Y	Y	Y	Y	Y	Y	Y	Y	Y	Y	Y	Y	Y	Y
	3.3	N	PN	N	N	N	N	N	Y	Y	NI	N	N	N	PN	N
	Judgement	Low	Low	Low	Low	Low	Low	Low	PN	PN	Low	Low	Low	Low	Low	Low
4. Deviations from intended interventions	4.1	N	N	N	N	N	N	PN	PN	PN	N	N	N	N	PN	N
	4.2	NA	NA	NA	NA	NA	NA	NA	NA	NA	NA	NA	NA	NA	NA	NA
	4.3	NA	NA	NA	NA	NA	NA	NA	NA	NA	NA	NA	NA	NA	NA	NA
	4.4	NA	NA	NA	NA	NA	NA	NA	NA	NA	NA	NA	NA	NA	NA	NA
	4.5	NA	NA	NA	NA	NA	NA	NA	NA	NA	NA	NA	NA	NA	NA	NA
	4.6	NA	NA	NA	NA	NA	NA	NA	NA	NA	NA	NA	NA	NA	NA	NA
	Judgement	Low	Low	Low	Low	Low	Low	Low	Low	Low	Low	Low	Low	Low	Low	Low
5. Missing data	5.1	PY	Y	Y	Y	Y	N	PY	NI	NI	Y	Y	Y	Y	NI	N
	5.2	NP	N	N	N	N	Y	PN	NI	NI	N	N	N	N	NI	N
	5.3	PN	N	N	N	N	N	PN	NI	NI	N	N	N	N	NI	N
	5.4	NA	NA	NA	NA	NA	PY	NA	NA	NA	NA	NA	NA	NA	NA	NA
	5.5	NA	NA	NA	NA	NA	N	NA	NA	NA	NA	NA	NA	NA	NA	NA
	Judgement	Low	Low	Low	Low	Low	Moderate	Low	NI	NI	Low	Low	Low	Low	NI	Low
6. Measurement of outcomes	6.1	PN	N	PN	N	PN	PN	PN	N	N	PY	PN	PN	N	N	N
	6.2	PY	NI	Y	N	Y	PY	NI	NI	NI	Y	N	N	PN	Y	NI
	6.3	Y	Y	Y	Y	Y	Y	Y	PY	PY	Y	Y	Y	Y	Y	Y
	6.4	N	N	N	N	N	N	PN	PN	PN	PN	N	N	N	PN	N
	Judgement	Moderate	Low	Moderate	Low	Moderate	Moderate	NI	NI	NI	Moderate	Low	Low	Low	Moderate	Low
7. Selection of the reported result	7.1	N	N	N	N	PN	N	PN	N	N	N	PN	PN	N	NI	N
	7.2	N	N	N	N	PN	N	PN	NI	NI	N	N	N	N	NI	N
	7.3	N	N	N	N	PN	N	PN	PN	PN	N	N	N	N	NI	N
	Judgement	Low	Low	Low	Low	Low	Low	Low	NI	NI	Low	Low	Low	Low	NI	Low
Overall	Judgement	Serious	Serious	Moderate	Low	Serious	Moderate	NI	Serious	Serious	Serious	Serious	Serious	Low	Moderate	Low

N, no; NA, not applicable; NI, no information; PN, probably no; PY, probably yes; Y, yes

Technological advancements in dentistry and orthodontics have increased treatment expectations of patients seeking orthodontic treatment, while being driven by both aesthetic and functional demands. The biomechanical background of orthodontic tooth movement with aligners has undergone rapid development during the last years. The aligner industry is an aspiring counterpart to standard conventional fixed-appliance orthodontic treatment.^22,33,53^ Aesthetic advantages and claims of increased comfort,^29^ easy application, and decreased treatment duration support aligner industries’ assertions and subsequently influence patient expectations.^36^

With the introduction of aligner use in clinical practice, reports have emerged about their potential advantages in terms of oral hygiene, dental and periodontal health.^1,33^ The specific target indices are the plaque index score (PI) and pocket probing depth (PPD) as well as the full-mouth bleeding score (FMBS),^3,26,34,51^ which are further exaggerated by the patient’s age and duration of orthodontic treatment.^3,9^ Importantly, critical factors for maintaining optimal oral hygiene levels during orthodontic treatment are patient cooperation, motivation, and personal knowledge about their periodontal health.^5,7,27^

In this respect, one might argue that improving the gingival and periodontal health indices might be expected during aligner treatment. It has been speculated that the part-time and removable nature of such appliances may result in potentially higher levels of oral hygiene maintenance, lowering the risk of developing gingivitis or tooth demineralisation.^1,6,26,31^ Conversely, awareness has been raised concerning oral microbiome and periodontal health status of patients undergoing treatment with aligners, mainly due to the ‘full-coverage’ effect of such appliances and adjuncts.^10,34,45,54^

Several reviews have been published lately, comparing aligner to fixed-appliance therapy; however, their focus has been somewhat variable, with specific interest on orthodontic treatment outcome,^42,43^ forces and moments generated by aligners^4,23^ and safety considerations.^24^ Furthermore, no comprehensive approach has been followed-up to date to review the existing evidence on oral health conditions overall, including dental and gingival-periodontal health of patients undergoing aligner treatment vs fixed-appliance therapy. The two available reviews^26,45^ focussed solely on periodontal health indices, with the most recent reporting a search strategy from almost four years ago.^26^ Since then, many primary studies have been published, with an increased dynamic being documented during the last 3 years.^2,8,11,32^

Therefore, the present systematic review aimed to answer the question: ‘Is aligner treatment for orthodontic tooth movement superior to the gold standard of fixed appliances with regard to oral hygiene status and, more specifically, the periodontal status and caries formation?’. The null hypothesis is that there is no difference between aligners and fixed appliances concerning oral hygiene maintenance during treatment.

## Materials and Methods

### Protocol and Reporting

Cochrane’s protocol was followed in this review, using Review Manager 5.4.1, the official software of Cochrane’s database (Review Manager [RevMan] computer program, version 5.4.1 Copenhagen: The Nordic Cochrane Centre, The Cochrane Collaboration, 2020). Furthermore, the reporting of this review followed the recommendations of the PRISMA statement.^37^ The protocol was registered with the Open Science Framework (https://osf.io/txgj6/).

### Search Strategy

An electronic search was conducted of the published and unpublished literature, separately, and by two examiners (EO, PF). The primary formal databases utilised in this study were MEDLINE via Pubmed, Scopus, Cochrane Central, and Cochrane Database for Systematic Reviews. Studies from the grey literature, defined as theses, dissertations, product reports, and unpublished studies, were found using ClinicalTrials.com, Open Grey, and ISRCTN. Hand searching was conducted in the retrieved literature for full-text evaluation of any additional articles with potential for inclusion. No filters were used. The search was performed on August 21, 2020 and updated on May 31, 2021. The entire search strategy for PubMed is presented in Appendix 1.

### Eligibility Criteria

Eligibility criteria for study selection were:

- Study design: Randomised controlled trials (RCTs), prospective clinical trials (PCTs), and observational studies were included in the review. Studies comparing at least two groups were considered. Specifically, these comprised full-arch treatment with orthodontic aligners either with fixed appliances or with a different type of orthodontic aligner (i.e. Invisalign vs clear aligners or removable appliances).
- Participants: All patients undergoing orthodontic treatment (no age or gender restriction).
- Intervention: orthodontic treatment (any) with aligners (any).
- Comparators: fixed-appliance orthodontic treatment, other aligner treatment/removable appliances.
- Outcome: oral hygiene measures, including but not confined to: gingival index (GI), plaque index (PI), bleeding on probing (BoP), probing depth (PD), clinical attachment loss (CAL), recession, the concentration of cariogenic and periodontal microflora in the surrounding tissues, as well as formation of incipient (i.e. white lesions) or advanced caries lesions.
- Exclusion criteria: Animal studies, case reports/series, non-clinical studies, and studies not performed in vivo were excluded. Studies without at least one control and one test group, studies including previously treated orthodontic patients, studies without comprehensive orthodontic management, and studies with ineligible results for this review were excluded. Only RCTs and prospective clinical studies were included in the quantitative data synthesis.

### Study Selection Process

The studies collected from all databases were cross-checked for the exclusion of duplicates. According to the study’s main characteristics of interest, titles and abstracts were screened independently by 3 reviewers (EO, PF, AT), with further exploration of the full text. Each reviewer forwarded the studies for inclusion and exclusion, according to eligibility criteria. Potential discrepancies were discussed among reviewers until a consensus was established. A fourth and fifth reviewer (CR, DK) were consulted when necessary to settle disagreements.

### Data Collection

Data were extracted and recorded in standardised piloted forms (Zotero 5.0.47, Corporation for Digital Scholarship; Vienna, VA, USA, and the Roy Rosenzweig Center for History and New Media; George Mason University, Fairfax, VA, USA). These forms included specific characteristics of the study (type, title, authors, abstract, publication, volume, issue, pages, date, series, series title, series text, journal abbreviation, language, DOI, URL, ISSN, short title, mean of access, archive, location in the archive, library catalogue, call number, date added, date modified). Data were extracted by three of the reviewers (EO, PF, AT) and re-examined by another two (CR, DK). Inconsistencies were discussed among reviewers until a consensus was reached.

### Risk of Bias in Individual Studies

The methodological quality of the studies was assessed by the Cochrane Risk of Bias tool 2.0 for Randomized Controlled Trials^49^ and the ROBINS-I (Risk of Bias in Non-randomized Studies – of Interventions) for controlled trials and observational studies.^48^

### Summary Measures and Data Synthesis

Quantitative syntheses of the studies’ findings were performed, if applicable, and after exploring heterogeneity levels across individual reports. Clinical heterogeneity was examined related to individual study settings as well as participants’ characteristics and eligibility criteria. Statistical heterogeneity was also assessed, visually first, via inspection of the confidence limits within the Forest plots, and also statistically using an I^2^ test, where a p-value < 0.10 was indicative of non-homogeneity. Random effects meta-analysis was conducted in view of the potential heterogeneity anticipated, under the DerSimonian and Laird variance estimator. Pooled estimates and 95% confidence intervals (95%CIs) were presented if two or more studies were deemed eligible for a single comparison. Prediction intervals were also computed, where applicable (at least 3 studies needed), in order to incorporate an assessment of a range of effects in future clinical settings. Effect measures were either mean differences (MD), or risk ratios (RR), depending on the nature of the retrieved outcome. Study authors were contacted for additional data requests if not all available information was provided within the published document.

### Risk of Bias across Studies

It was planned to explore publication bias through standard funnel plots and Egger’s regression test, if applicable.^13^

### Additional Analyses

Sensitivity analyses were considered, if applicable, to explore and isolate the effect of studies with serious/critical/high risk of bias on the overall impact, if studies of both serious/critical/high or low risk of bias were ultimately included in the quantitative synthesis.

### Assessment of the Quality of the Evidence

Grading of Recommendations Assessment, Development, and Evaluation (GRADE) was implemented to assess the overall quality of the evidence as formulated by the question, treatments, and outcomes for evaluation. According to GRADE, the overall body of evidence was rated as high, moderate, low, and very low. Assessment of the body of evidence primarily involves assessment of study design. Assessment is made on the following domains: risk of bias, inconsistency, indirectness, imprecision, and publication bias. For the first 4 domains, the quality of evidence may be downgraded based on either ‘serious’ or ‘very serious’ risks (1 or 2 levels respectively); publication bias may either be suspected or undetected. For non-randomised/observational designs in particular, which theoretically start from a ‘low’ level of evidence, the possibilities for an upgrade are as follows: a large or very large effect, plausible residual confounding that may alter the effect, or a dose-response gradient. The level of evidence may be upgraded by 1 or 2 levels (large effect), or 1 level (plausible confounding, dose-response gradient).^20^

## Results

### Search Details

**Fig 1:**
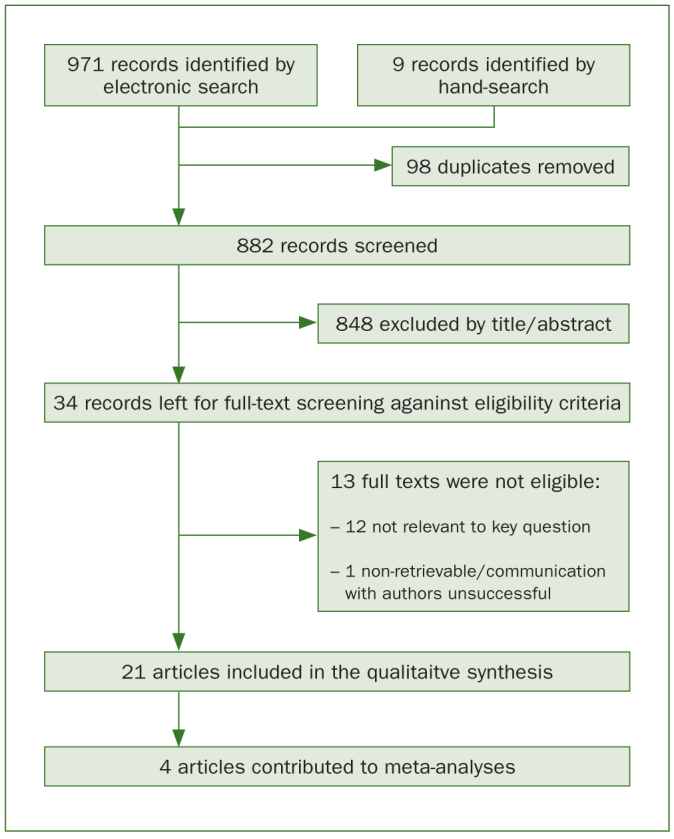
Flow diagram of study selection and inclusion.

The complete study selection process, from searching to inclusion, is presented in Fig 1. From an initial hit of 971 articles, after additional hand searching and duplicate removal (882 unique records screened), 21 articles passed the full-text screening process and were included in the qualitative synthesis. Of those, 4 qualified^28,30,39,40^ for quantitative syntheses (meta-analyses).

### Study Design and Characteristics

Detailed characteristics of included studies and findings of the data extraction process are presented in Table 1. Of a total of 21 publications, the study design breakdown was as follows: 6 RCTs, 11 prospective clinical trials (PCTs), 3 retrospective cohorts, and one cross-sectional study. Publication dates of these studies varied from 2005 to 2020, with the majority being published within the years 2018 and 2020 (11/21; 52.4%).

**Table 1:** Characteristics of included studies

Authors (year), type	Participants	Intervention	Comparator	Outcomes	Additional information
Abbate et al (2015), RCT	50 teenagers (10–18) treatment with fixed brackets (n = 25) Treatment with Invisalign aligners (n = 25)	Invisalign	Fixed labial brackets	Full mouth plaque score Full mouth bleeding score Plaque index Bleeding on probing Probing depth Oral hygiene compliance	3 dropouts on aligner group Timeline: 3, 6, 12 months
Albhaisi et al (2020), RCT	49 participants, 39 female, 10 male, 21.25±3, range 17–24	Clear aligners	Fixed labial appliances	Fluorescence loss (ΔF) Number of newly developed lesions Deepest point in the lesion (ΔF_Max_) Lesion area (pixels) and plaque surface area (DR30) were measured as secondary outcomes. Fluorescence was assessed using QLF.	Timeline: 3 months (use of attachments)
Azaripour et al (2015), retrospective cohort	100 participants: 50 with Invisalign (11 males and 39 females, mean age 31.9 ± 13.6 years; range: 12–61 years) and 50 with fixed appliances (34 females and 16 males, mean age 16.3 ± 6.9 years old; range 11–61 years)	Invisalign	Fixed labial appliances	API SPI GI	Timeline: 12 months (at least 6 months in treatment)
Bushang et al (2018), retrospective cohort	450 participants, aligners (30.4 ± 14 years), fixed (29.2 ± 11.5 years), aligners (64% females, 36% males), fixed (63% females, 37% males)	Clear aligners	Fixed labial appliances	OH WSLs	85% of the aligner cases and 48% of the fixed cases were taken from private practice Timeline: throughout treatment duration
Chhibber et al (2017), RCT	71 participants 41 boys 30 girls. Mean ages of participants 16.56 + 3.99 in CLA group (27 participants), 15.39 + 3.54 in SLB group (22 participants), and 14.56 ± 3.92 in ELB group (22 participants)	Invisalign	Self-ligating brackets Elastomeric ligating brackets	PI GI PBI	Three in the CLA group, 5 in the SLB group, and 2 in the ELB group were completely lost to follow-up, and 2 in the SLB group and 1 in the ELB group were partially followed-up. Timeline: 9 and 18 months
Dallel et al (2020), PCT	112 participants, 10–20 years of age, 49.1% females, 50.9% males	Invisalign	Fixed labial appliances Andresen type II activator	Volume and salivary flow Biochemical parameter measurement Trolox equivalent antioxidant capacity WSLs	Timeline: 1 and 9 months
Gujar et al (2019), RCT	40 participants (age 12–32 years; mean 28±4 years), 23 females, 17 males	Clear aligners (probably Invisalign, but not clearly specified)	Fixed labial appliances	Cytokine levels PI GI POB	Timeline: 21 days
Gujar et al (2020), PCT	60 participants, 11–29 years of age	Invisalign	Fixed labial appliances Fixed lingual appliances	Microbial level changes using checkerboard DNA-DNA hybridisation	Samples were collected only from the maxillary arch Timeline: 30 days
Han et al (2015), retrospective cohort	35 participants with periodontitis, 21 females, 14 males, mean age 52.97 ± 9.42 years, range 35–74	Clear aligners	Fixed labial appliances	PI reduction GI reduction PD reduction Bone level improvement	The male:female ratio was statistically significantly different between the 2 groups 1 patient smoked CAT was used for severely mobile or labial inclined teeth Timeline: throughout treatment duration
Karkhanetci et al (2013), PCT	42 participants, FA group: 16 females, 6 males, 34 ± 7.18 years of age, range 18–44. Invisalign group: 12 females, 8 males, 28 ± 6.86 years of age, range 18–44	Invisalign	Fixed labial appliances	PI GI BoP PPD BANA (secondary outcome)	Modest sample size 17% attrition rate Timeline: 1.5, 6, 12 months
Levrini et al (2013), RCT	30 adults (10 Invisalign, 10 fixed appliances, 10 no intervention)(9 males, 21 females, aged 25.1 ± 4.6)	Invisalign	Fixed appliances, no intervention	PI PD BOP Compliance with OH Subgingival microbial samples	Timeline: 1 and 3 months
Levrini et al (2015), RCT	77 participants (5 male Invisalign, 18 male fixed appliances, 2 control, 27 male Invisalign, 17 male fixed appliances, 8 control) age range 16–60 years	Invisalign	Fixed appliances, no intervention	PI PD BOP Biofilm mass Periodontal pathogens (PCR)	Timeline: 1 and 3 months
Madariaga et al (2020), PCT	40 participants with permanent dentition (26 females, 14 males) mean age 27.6 ± 12.6 years, 20 treated with aligners 20 with fixed appliances	Clear aligners	Fixed appliances	PD PI BOP REC (gingival recession)	Timeline: 3 months
Miethke and Vogt (2005), PCT	60 participants (43 female, 17 male, 30 Invisalign, 30 fixed appliances) Mean age 30.1 years, range 18–51	Invisalign	Fixed appliances	Modified GI Modified PI Modified PBI SPI	It is possible, though not stated, that at least 1 patient was treated with both brackets and aligners concurrently Timeline: after 1, 2, and 3 months (patients in treatment for at least 6 months)
Miethke and Brauner (2007), PCT	60 participants 30 with Invisalign, 30 with fixed lingual appliances. Age information in the fixed appliances group 16–48 years, mean age: 39.6 years	Invisalign	Fixed lingual appliances	Modified GI Modified PI Modified PBI SPI	Invisalign group was used in a previous study (Miethke et al, 2005). Some participants were recruited from private practices Timeline: after 1, 2, 3 months (patients in treatment for at least 6 months)
Mulla Issa et al (2020), cross sectional	80 participants, 50% males, 50% females, mean age: 27±5.8 years; range: 23–29 years	Clear aligners (Angle Align, China / Invisalign)	Fixed labial appliances with conventional brackets (a), ceramic brackets (b), self-ligating brackets (c)	PI GI GBI SBI PBI BPE BOP	Timeline: at least 6 months in treatment, recorded once
Mummolo et al (2020a), PCT	90 participants (30 had Invisalign, mean age 21.5±1.5 years, 30 had fixed appliances, mean age 23.3±1.6 years, 30 had removable positioners, mean age 18.2 ±1.5 years)	Invisalign	Fixed appliances, removable positioners (RP) (Occlus-o-Guide)	Salivary concentrations of *S. mutans* and lactobacilli (CRT bacteria) PI	Timeline: 3 and 6 months
Mummolo et al (2020b), PCT	80 participants (40 [16 females, 24 males] Invisalign with mean age 20.4±1.7 years; 40 [18 females, 22 males] fixed appliances with mean age 21.3±1.7,)	Invisalign	Fixed labial appliances	PI Salivary flow (CRT prevention system) Buffering power of saliva (CRT buffer) Salivary levels of *S. mutans* and lactobacilli (CRT bacteria)	Timeline: 3 and 6 months
Sifakakis et al (2018), PCT	30 participants, 17 females, 13 males, mean age 13.8 years, range 12–18 years	Clear aligners	Fixed labial appliances	Simplified PI Simplified GI DMFT qPCR (for cariogenic bacteria)	Self-ligating fixed appliances Timeline: 2 weeks, 1 month
Srinath et al (2016), PCT	46 participants. Fixed appliances group: 18 women and 8 men with a mean age of 34 ± 7.18 years, range of 22–44 years. Aligners group: 8 men and 12 women, mean age: 35 ± 6.86 years, range: 18–38 years	Clear aligners	Fixed appliances	GI, PD, BOP	Timeline: 6 weeks, 6 months, 12 months
Wang et al (2019), PCT	26 participants, 20–25 years	Invisalign	Fixed labial appliances	16S rRNA gene identified through pyrosequencing	Only 5 subjects were selected randomly from each group for high-throughput pyrosequencing analysis Timeline: one single saliva sample collection (at least 6 months in treatment)

API: approximal bleeding index; BPE: basic periodontal examination index; BOP: bleeding on probing; CAT: clear aligner technique; CLA: clear aligners; DMFT: decayed, missing, filled teeth; ELB: elastomeric ligated brackets; FA: fixed appliances; GBI: gingival bleeding index; GI: gingival index; OH: oral hygiene; PBI: papillary bleeding index; PCT: prospective clinical trial; PI: plaque index; PPD: pocket probing depth; REC: gingival recession; RCT: randomised controlled trial; SBI: sulcus bleeding index; SLB: self-ligating brackets.

Fifteen studies examined the effects of treatment in adult patients, with one of those in participants with chronic periodontitis.^21^ Four involved only teen/adolescent participants, while two included a wide range of ages including both teenagers and adults. Sample sizes ranged from 26 to 112 patients for RCTs and prospective clinical trials, while those of retrospective and cross-sectional studies were between 35 and 450.

In 13 of 21 studies, the intervention group comprised Invisalign (Align Technology; San Jose, CA, USA) clear-aligner treatment, while in the rest of the studies, thermoformed clear-aligner appliances were used. Traditional labial fixed-appliance treatment was provided in comparator groups in 20 out of 21 studies, with one including patients with both labial and lingual multi-bracket appliances and another one in which the comparator group had lingual fixed appliances. A number of outcomes related to oral and periodontal hygiene were recorded. Briefly, plaque indices, bleeding scores, probing depth, and gingival indices were the most frequently recorded outcomes. In addition, salivary concentrations of microbial and cariogenic bacteria, such as *S. mutans* and lactobacilli were reported. Outcomes related to carious lesions and lesion characteristics, such as fluorescence loss or lesion area, were also recorded. The range of follow-up times for outcome assessment was between 1 month after initiation of treatment and 18 months; however, two retrospective studies^8,21^ reported outcomes pertaining to evaluation after completion of orthodontic treatment, considering the whole treatment duration.

### Risk of Bias within Studies

The risk of bias for the RCTs included in the present systematic review ranged from low to high overall. The latter primarily pertained to suboptimal reporting of randomisation practices in 2 of the studies; a classification of high risk of bias was decided in this respect, as identified problems with randomisation practices would potentially induce selection bias. Identified issues with inadequate reporting were also related to deviations from intended interventions and missing outcome data (Table 2, supplementary Table 1).

**Table 2:** Risk of bias of included randomised controlled trials with the RoB 2.0 tool

Study	Randomisation	Deviations from intended interventions	Missing outcome data	Measurement of the outcome	Selection of the reported result	Overall
Abbate et al, 2015	Low	Low	Low	Some concerns	Some concerns	Some concerns
Albhaisi et al, 2020	Low	Some concerns	Low	Low	Low	Some concerns
Chhibber et al, 2017	Low	Low	Some concerns	Low	Low	Some concerns
Gujar et al, 2019	Low	Low	Low	Low	Low	Low
Levrini et al, 2013	High	Low	Low	Low	Low	High
Levrini et al, 2015	High	Some concerns	High	Low	Low	High

Among the non-randomised studies, those that were not prospective were deemed to be at serious risk of bias, mainly due to confounding issues or bias related to selection of participants to be included in the studies. With regard to prospective clinical trials, studies were categorised within the range of low to serious risk of bias. The most severely impacted domains were undetected confounding and subsequent risk for selection bias, while also the risk of detection bias and systematic differences in the measurement of the outcomes could not be neglected (Table 3, supplementary Table 2).

**Table 3:** Risk of bias of included non-randomised studies according to the ROBINS-I tool

	Bias due to / in…							
	Confounding	Selection of participants for the study	Classification of interventions	Deviations from intended interventions	Missing data	Measurement of outcomes	Selection of the reported result	Overall
Azaripour et al, 2015	Serious	Serious	Low	Low	Low	Moderate	Low	Serious
Buschang et al, 2018	Serious	Serious	Low	Low	Low	Low	Low	Serious
Dallel et al, 2020	Moderate	Low	Low	Low	Low	Moderate	Low	Moderate
Gujar et al, 2020	Low	Low	Low	Low	Low	Low	Low	Low
Han et al, 2015	Serious	Serious	Low	Low	Low	Moderate	Low	Serious
Karkhanetci et al, 2013	Moderate	Low	Low	Low	Moderate	Moderate	Low	Moderate
Madariaga et al, 2020	No Information	No Information	Low	Low	Low	No Information	Low	No Information
Miethke et al 2005	Serious	No Information	Low	Low	No Information	No Information	No Information	Serious
Miethke et al, 2007	Serious	No Information	Low	Low	No Information	No Information	No Information	Serious
Mulla Issa et al, 2020	Serious	Serious	Low	Low	Low	Moderate	Low	Serious
Mummolo et al, 2020a	Serious	Low	Low	Low	Low	Low	Low	Serious
Mummolo et al, 2020b	Serious	Low	Low	Low	Low	Low	Low	Serious
Sifakakis et al, 2018	Moderate	Low	Low	Low	Low	Low	Low	Moderate
Srinath et al, 2016	No Information	No Information	Low	Low	No Information	Moderate	No Information	Moderate
Wang et al, 2019	Low	Low	Low	Low	Low	Low	Low	Low

### Effects of Interventions, Meta-Analysis and Additional Analyses

**Fig 2:**
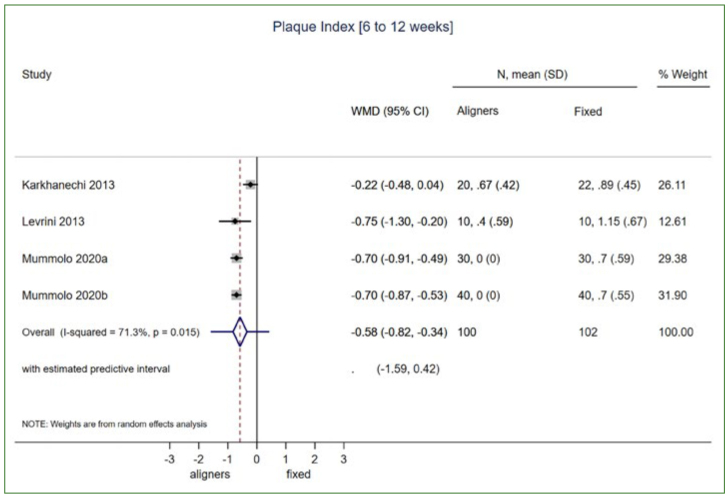
Random effects meta-analysis for summary mean difference (with 95% CI) in overall plaque index (PI) scores in adults undergoing aligner orthodontic treatment compared to standard fixed appliances, within the first 6 to 12 weeks of treatment initiation.

Overall, 4 studies were included in the meta-analysis, all related to periodontal outcomes (Table 4). Synthesised data were available only for adult patients undergoing treatment with either aligners of fixed appliances. Again, synthesised data (i.e. ≥ 2 studies) of the 4 eligible studies involved only patients treated with Invisalign (Align Technology). Patients undergoing aligner orthodontic treatment presented summary PI scores 0.58 lower than those treated with standard fixed appliances, within the first 6 to 12 weeks of treatment (4 studies: MD: -0.58; 95%CI: -0.82, -0.34; p < 0.001; I^2^: 71.3%; prediction interval: -1.59, 0.42; Fig 2). This finding was supported by a decreased PD of 0.42 mm in aligner patients (2 studies: MD: -042; 95%CI: -0.71, -0.12; p = 0.006; I^2^: 85.8%). In contrast, inflammation and bleeding indices, such as BOP and GI, did not reveal statistically significant differences between the aligner and fixed-appliance treatment groups during the same timeline of 6 to 12 weeks in adult patients (Table 4).

**Table 4:** Results of meta-analyses and single study estimates related to periodontal outcomes (aligners vs fixed appliances)

Synthesis	No. of studies	Effect measure (MD)	95% CI	p-value	I^2^ (%)	Tau-squared (T^2^)
Adults						
PI (6 to 12 weeks)^1^	4	-0.58	-0.82, -0.34	<0.001	71.3	0.04
BOP (6 to 12 weeks)^2^	2	-0.26	-0.77, 0.26	0.33	85.8	0.12
PD (6 to 12 weeks)^2^	2	-0.42	-0.71, -0.12	0.006	34.7	0.02
GI (6 to 12 weeks)^3^	1	-0.10	-0.35, 015	0.43	–	–
Adolescents						
PI (18 months)^4^	1	-0.40	-0.77, -0.03	0.04	–	–
s-PI (1 month)^5^	1	-14.78	-16.74, -12.82	<0.001	–	–
GI (18 months)^4^	1	-0.57	-0.93, -0.21	0.002	–	–
s-GI (1 month)^5^	1	-8.46	-10.47, -6.45	<0.001	–	–
FMPS (12 months)^6^	1	-43.48	-47.65, -39.31	<0.001	–	–
FMBS (12 months)^6^	1	-20.44	-22.98, -17.90	<0.001	–	–

BOP, bleeding on probing; CI, confidence interval; FMBS, fullmouth bleeding score; FMPS, fullmouth plaque score; GI, gingival index; MD, mean difference; PI, plaque Index; PD, pocket depth; s-PI, simplified plaque index; s-GI, simplified gingival index. ^1^Karkhanechi et al 2013, Levrini et al 2013, Mummolo et al 2020a, Mummolo et al 2020b; ^2^Karkhanechi et al 2013, Levrini et al 2013; ^3^Karkhanechi et al 2013; ^4^Chhibber et al 2018; ^5^Sifakakis et al 2018; ^6^Abbate et al 2015.

Based on the available studies, only single study estimates could be recorded in relation to adolescent patients, while no mathematical synthesis was possible. Based on these estimates, plaque and gingival/ bleeding scores appeared lower for teenagers treated with aligners. According to the single study^10^ with the longer follow-up period (i.e. 18 months), PI scores for the aligner group was 0.40 lower than the corresponding figure for fixed appliances (1 study: MD: -0.40; 95%CI: -0.77, -0.03; p = 0.04); additionally, GI was 0.57 lower in aligners compared to fixed appliances (1 study: MD: -0.57; 95%CI: -0.93, -0.21; p = 0.002). Results from the other two studies^28,46^ with shorter follow-up periods confirmed this (Table 4).

Regarding microbiological parameters in adult patients, 4 meta-analyses, each comprising 2 studies from the same group, were deemed possible (Table 5). The risk of *S. mutans* presence in detectable concentrations (colony forming units, CFU > 10^5^) for a period of 3 to 6 months after initiation of treatment was 74% to 93% lower in aligner patients (2 studies/3 months: RR: 0.07; 95%CI: 0.01, 0.49; p = 0.008; I^2^ = 0.0%; 2 studies/6 months: RR: 0.22; 95%CI: 0.10, 0.51; p = 0.001; I^2^ = 0.0%). Likewise, the risk of lactobacilli occurrence (CFU > 10^5^) in a similar time interval was 81% to 91% lower in aligner groups (2 studies/3 months: RR: 0.09; 95%CI: 0.02, 0.47; p = 0.004; I^2^ = 0.0%; 2 studies/ 6 months: RR: 0.19; 95%CI: 0.08, 0.45; p < 0.001; I^2^ = 56.9%) (Table 5).

**Table 5:** Results of meta-analyses and single study estimates, related to microbiologic parameters (aligners vs fixed appliances)

Synthesis	No. of studies	Effect measure	95% CI	p-value	I^2^ (%)	Tau-squared (T^2^)
Adults						
*S. mutans* (CFU > 10^5^, 3 months)^1^	2	RR: 0.07	0.01, 0.49	0.008	0.0	–
*S. mutans* (CFU > 10^5^, 6 months)^1^	2	RR: 0.22	0.10, 0.51	0.001	0.0	–
Lactobacilli (CFU > 10^5^, 3 months)^1^	2	RR: 0.09	0.02, 0.47	0.004	0.0	–
Lactobacilli (CFU > 10^5^, 6 months)^1^	2	RR: 0.19	0.08, 0.45	<0.001	56.9	–
Adolescents						
*S. mutans* (presence, 1 month)^2^	1	RR: 0.86	0.64, 1.14	0.29	–	–
*S. mutans* (counts: 5th root, 1 month)^2^	1	MD: -2.22	-6.82, 2.38	0.34	–	–

CFU: colony forming units; CI: confidence intervals; MD: mean difference; RR: risk ratio. ^1^Mummolo et al 2020a, Mummolo et al 2020b; 2 Sifakakis et al 2018.

In contrast, in adolescents, evidence from a single study estimate^46^ on the risk of *S. mutans* colonisation did not showe a statistically significant difference between the groups under examination. However, these findings were derived from a short-term evaluation of 1 month after initiation of treatment (1 study: aligners vs fixed appliances, RR: 0.86; 95%CI: 0.64, 1.14; p = 0.29) (Table 5).

Data related to incipient caries/WSLs were derived only from single-study estimates of two recently published studies.^2,11^ According to these findings, adult patients presented a 28% lower risk for the development of WSLs (on tooth level) when assessed over a 3-month period (1 study, RR: 0.72; 95%CI: 0.60, 0.86; p < 0.001). Moreover, average fluorescence loss, denoting mineral tissue loss, was lower in aligner-treated patients (1 study, mean difference [MD]: -1.40; 95%CI: -2.15, -0.65; p < 0.001). Interestingly, however, when the lesion area (in pixels) was examined, patients treated with aligners presented an increased area of decalcification (1 study, MD: 80.50; 95%CI: 60.52, 100.48; p < 0.001). In contrast, no difference in the formation of WSLs (patient level) was detected in adolescent patients between the two different orthodontic treatment techniques (1 study, aligners vs fixed appliances, RR: 0.33; 95%CI: 0.10, 1.04; p = 0.06) (Table 6).

**Table 6:** Results of single study estimates, related to WSLs (aligners vs fixed appliance)

Synthesis	No. of studies	Effect measure	95% CI	p-value	I^2^ (%)	Tau-squared (T^2^)
Adults						
WSLs (tooth level/3 months)^1^	1	RR: 0.72	0.60, 0.86	<0.001	–	–
Average fluorescence loss (ΔF%), 3 months^1^	1	MD: -1.40	-2.15, -0.65	<0.001	–	–
Lesion area (pixels), 3 months^1^	1	MD: 80.50	60.52, 100.48	<0.001	–	–
Adolescents						
WSLs (patient level/ 9 months)^2^	1	RR: 0.33	0.10, 1.04	0.06	–	–

CI: confidence interval; MD: mean difference; RR: risk ratio; WSL: white spot lesion. ^1^Albhaisi et al, 2020; ^2^Dallel et al, 2020.

Further sensitivity analysis or publication bias assessment was ultimately not conducted due to the paucity of existing studies contributing to the quantitative synthesis.

### Quality of Evidence

**Fig 3:**
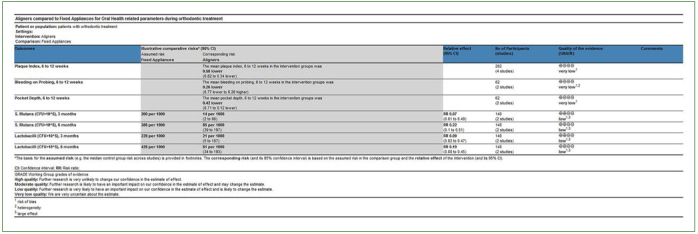
Assessment of the quality of the available evidence based on synthesised data, according to GRADE

The quality of the existing evidence for the outcomes assessed after data synthesis ranged from very low to low overall, based on a limited number of pooled studies. Specifically, for PI and PD indices, the quality of the evidence was recorded as very low, based on a combination of randomised and non-randomised (prospective) studies and due to risk of bias suspected for contributing studies. In addition, for BOP, the certainty of the evidence was downgraded for heterogeneity reasons as well. For microbiological parameters, and based on the syntheses of non-randomised (prospective) data, the quality of evidence was downgraded due to problems with the internal validity of the contributing studies, while it was upgraded as a result of identification of a large pooled effect. As such, the certainty of evidence was ultimately rated as low overall (Fig 3).

## Discussion

### Findings in Context and Previous Research

Aligner treatment has become a popular option among patients, thanks to aesthetic considerations and perceived ease and comfort of the therapeutic procedures. Maintaining an acceptable level of oral hygiene is of paramount importance for the duration of treatment to avoid any adverse effects related to loss of tooth integrity and periodontal health.

While acknowledging all limitations of the present study and the synthesised data, there is some evidence that orthodontic treatment with aligners might prove beneficial at least in the short-term, especially for adult patients. The present study is the first systematic review to consider all contemporary evidence on oral hygiene parameters related to orthodontic treatment with aligners, including periodontal parameters, microbiological parameters as well as incipient caries/white spot lesions. It thus constitutes a global and comprehensive evaluation of the efficacy of contemporary orthodontic appliances in helping maintain high levels of oral hygiene during treatment.

Previous studies in the field of oral hygiene and competing intervention procedures for orthodontic tooth movement have either shown a scarcity of evidence, being based on very few early reports up to 2015,^45^ or found clear aligner treatment to be more effective. The evidence was still heterogeneous and not of high quality, with a specific focus on periodontal health.^26^ Jiang et al^16^ concluded superior periodontal health status based on evidence from PI and GI, although the quality of the evidence was not high, mainly due to the risk of bias and inconsistency of the results. It is noteworthy that increased variability in synthesised data contributed to the pooled estimate concerning types of study designs included in this earlier review, as well as follow-up times and patient ages.^17^

Based on our findings from a short-term evaluation period (up to 6 months under treatment) mainly concerning adult patients, plaque scores along with microbial counts related to tooth decay demonstrate that aligner treatment may qualify as a treatment option that potentially offers improved oral hygiene status. However, it may be argued that such findings cannot be directly related to permanent effects on the periodontal apparatus or tooth integrity of a patient undergoing orthodontic treatment with standard fixed appliances. In addition, the clinical implications of the identified differences might be negligible or constitute a somewhat fragile and transient component. In support of that, and based on the prediction interval related to the meta-analysis of PI scores, the expected effects of aligner treatment in future trials or settings might document improved oral hygiene status – or not.

Following the initial use of orthodontic aligner and their utilisation in more complex orthodontic cases, thanks to the latest technological developments, attachment grips bonded onto the enamel have been introduced, with growing and large-scale applications in practice.^16,24^ Such adjuncts may vary in dimensions and morphology, ranging from 2 mm to 5 mm, and also demonstrate increased width, often exceeding 1 mm.^12^ Such variations are usually required to increase aligner retention to the dentition while also facilitatiing force generation and induction of 3-dimensional movements. The shortcomings of the wide use of bonded grips and bulky composites on tooth enamel are associated with an increased potential for food, plaque, and microbial accumulation, as well as biofilm formation,^50^ thus compromising the oral health of the treated individual. Intraoral aging of both the aligner material and the attachment have been linked to alteration of material properties, which may, in turn, influence oral hygiene.^41^ Plaque can accumulate very soon after initial bonding, while a crucial determinant of biofilm adhesion ability is surface roughness.^44^ A rough surface enables bacterial adhesion to the material, forming a relatively protected environment for microbes against oral hygiene measures.^14^ A recent in vitro study demonstrated no differences between different types of aligner materials in terms of initial bacterial attachment levels and biofilm formation with metal brackets.^50^ Only one of the studies included in the present systematic review reported on the use of attachments.^2^ That study concluded that at least within the first 3 months of treatment, both fixed appliances and aligners induced incipient caries in the form of WSLs, although the WSLs differed. Orthodontic aligners were associated with shallower lesions with a larger area, which were mainly attributed to the bulky shape of the attachments. In contrast, patients treated with fixed appliances developed smaller lesions but with increased mineral loss and sites of decalcified tissue. Future studies should be designed for a more in-depth assessment of the influence of different types and shapes of attachment grips on enamel and tooth integrity.

Considering the synthesised data, indices related to periodontal inflammation, such as BOP in adults, did not strictly follow the findings from microbiological and plaque-related parameters. This, in turn, might indicate the reversible nature of the initial disruption of oral hygiene parameters shortly after the beginning of orthodontic treatment, at least for adult patients. On the other hand, findings from single studies examining the response of adolescents to orthodontic appliances confirm a more thorough short-term disruption of oral health parameters; thus, certain groups of patients might be considered more prone to limited compliance with oral hygiene measures and qualify as high-susceptibility patients during standard-appliance orthodontic treatment, at least in the short term. Apparently, additional measures of oral hygiene reinforcement in adolescent patients should be considered.^25^ In contrast, another single study reported no statistically significant differences in periodontal parameters related to fixed vs aligner therapy, indicating better oral-hygiene maintenance in such patients.^21^ However, in this respect, further and more comprehensive studies are needed to strengthen the available evidence.

Clinically, appliance type and treatment strategy for orthodontic tooth movement should be selected to optimise treatment outcome and safety, bearing in mind the long-term nature of orthodontic treatment and the retention period. Recently, it has been argued that orthodontic treatment with aligners – compared to gold-standard fixed appliances – in adult patients yields less effective treatment outcomes in terms of achieving occlusal goals.^42,43^ Hence, one should carefully weigh clinical outcomes, potential temporary adverse effects during treatment, treatment duration, and patient values before treatment initiation, ensuring that the patient is well-informed and consulted about treatment planning.^15^

### Strengths and Limitations

The present systematic review and meta-analysis provide the current state of evidence regarding oral hygiene parameters in general and during orthodontic treatment, comparing traditional fixed appliances with contemporary orthodontic aligners. It was performed strictly following prospective protocol development and registration, while an unconditional search strategy was applied to both published and unpublished literature for study identification and selection. Quantitative syntheses, risk of bias assessment, and evaluation of the certainty of the evidence were performed in line with reporting guidelines. Heterogeneity problems were acknowledged, and populations of different ages were examined separately, as these may demonstrate different mentalities in relation to oral hygiene measures and parameters. Pooled estimates were solely based on prospectively collected data, to ensure bias elimination.

However, limitations do exist. First and foremost are the quality and certainty of the evidence identified, although this is chiefly related to the primary studies included in the review. In addition, a relatively small number of studies were included in the quantitative synthesis. This may have allowed imprecision in the pooled estimates overall, while no additional analyses were conducted due to the scarcity of studies eligible for syntheses. Moreover, no follow-up time longer than 6 months of treatment could be included in the meta-analyses, given the available primary data and the heterogeneity of individual study settings.

## Conclusions

Aligner orthodontic therapy is associated with better oral hygiene levels in the short term; however, this is not corroborated by a high level of certainty of the available evidence. Therefore, any extrapolation to contemporary aligner techniques and adjuncts, such as attachment grips, is only speculative.

## Appendix 1 Search strategy for Medline (via PubMed)

PubMed (MEDLINE)

All Fields

Date: May 31st, 2021

Filters: none

1. Orthodontic aligner
2. Orthodontic aligner*
3. Invisalign
4. Clear orthodontic aligner
5. Thermoplastic orthodontic aligner
6. Vacuum-formed orthodontic aligner
7. Vacuum-formed orthodontic aligner
8. 1 OR 2 OR 3 OR 4 OR 5 OR 6 OR 7
9. Gingival index
10. Plaque index
11. Early caries
12. Incipient caries
13. Caries
14. Tooth decay
15. Bleeding
16. Oral hygiene
17. Periodontal health
18. Recession
19. Probing depth
20. *S. mutans*
21. *Streptococcus mutans*
22. Lactobacilli
23. Lactobacillus
24. Oral microbiome
25. 9 OR 10 OR 11 OR 12 OR 13 OR 14 OR 15 OR 16 OR 17 OR 18 OR 19 OR 20 OR 21 OR 22 OR 23 OR 24
26. 8 AND 25
